# Description of full-range strain hardening behavior of steels

**DOI:** 10.1186/s40064-016-2998-3

**Published:** 2016-08-11

**Authors:** Tao Li, Jinyang Zheng, Zhiwei Chen

**Affiliations:** 1 Institute of Process Equipment, Zhejiang University, Hangzhou, 310027 Zhejiang People’s Republic of China; 2 Chinese Standardization Committee on Boilers and Pressure Vessels, Beijing, 100029 People’s Republic of China; 3 High-Pressure Process Equipment and Safety Engineering Research Center of Ministry of Education, Zhejiang University, Hangzhou, 310027 Zhejiang People’s Republic of China; 4 The State Key Laboratory of Fluid Power Transmission and Control, Zhejiang University, Hangzhou, 310027 Zhejiang People’s Republic of China

**Keywords:** Strain hardening behavior, Stress strain curve, Plastic deformation

## Abstract

Mathematical expression describing plastic behavior of steels allows the execution of parametric studies for many purposes. Various formulas have been developed to characterize stress strain curves of steels. However, most of those formulas failed to describe accurately the strain hardening behavior of steels in the full range which shows various distinct stages. For this purpose, a new formula is developed based on the well-known Ramberg–Osgood formula to describe the full range strain hardening behavior of steels. Test results of all the six types of steels show a three-stage strain hardening behavior. The proposed formula can describe such behavior accurately in the full range using a single expression. The parameters of the formula can be obtained directly and easily through linear regression analysis. Excellent agreements with the test data are observed for all the steels tested. Furthermore, other formulas such as Ludwigson formula, Gardner formula, UGent formula are also applied for comparison. Finally, the proposed formula is considered to have wide suitability and high accuracy for all the steels tested.

## Background

The description of strain hardening behavior of materials using mathematical expression has been the subject of numerous investigations for many years. Strain hardening response of materials is usually characterized indirectly by the true stress–strain curves obtained from tensile tests. Typically, the strain hardening rate can be calculated numerically from the curves and plotted against strain (or stress). It is now well established that the hardening rate of crystals may be divided into various distinct stages (Nabarro et al. 1964; Asgari et al. 1997; Chinh et al. 2004), typically three stages, labeled Stage I, Stage II and Stage III (Kuhlmann-Wilsdorf 1985). The stages of polycrystalline steels are much less evident than those of the single crystal (Reedhill et al. 1973). Therefore, some forms of analysis are normally to describe the strain hardening behavior of steels. For this purpose, the Ramberg–Osgood formula (Ramberg and Osgood 1943) has been used widely for steels in various engineering fields. However, this formula is inherently deficient to describe the strain hardening behavior of steels in the full range.

Distinct stages strain hardening behavior has been observed in various types of steels (Jha et al. 1987; Nie et al. 2012; Umemoto et al. 2000; Tomita and Okabayashi 1985; Atkinson 1979; Kalidindi 1998; Saha et al. 2007). Many formulas were designed to describe the full-range hardening and some material-specific formulas have been proposed for stainless steels (Rasmussen 2003; Gardner and Nethercot 2004a, b; Abdella 2006; Quach et al. 2008; Arrayago et al. 2015), TRIP steels (Tomita and Iwamoto 1995), high strength steels (Gardner and Ashraf 2006) and pipeline steels (Hertelé et al. 2012a, b). Although excellent agreement has been provided for specific materials, the formulas have difficulty being adopted for other materials. Additionally, it should be noted that the strain hardening behavior involves a complex interaction among various factors. At the microscale, this aspect of plastic deformation is intrinsically coupled with all other aspects of plastic deformation such as development of preferred lattice orientations, formation of sub-grains, and formation of local shear bands (Wilson 1974). For austenitic steels and TRIP steels, the microstructural phase transformation from austenite to martensite also has a great effect on the plastic deformation. (Leblond et al. 1986a, b; Hallberg et al. 2007; Santacreu et al. 2006; Post et al. 2008; Stringfellow et al. 1992; Bhattacharyya and Weng 1994; Diani et al. 1995; Miller and McDowell 1996; Papatriantafillou et al. 2006; Turteltaub and Suiker 2005; Beese and Mohr 2012; Iwamoto and Tsuta 2000). This has been actively studied for decades. Therefore, it is virtually impossible to develop a complete understanding (Chinh et al. 2004) of the behavior, and no unified theory on the physically based functional description has been found (Cleri 2005). Most of these formulas to describe the strain hardening behavior of steel are purely empirical descriptions.

The purpose of this paper is to present a mathematical description of the full-range strain hardening behavior for steels with smooth, gradual onset of yielding. Note that many mathematical descriptions have already existed, an overview of existing stress–strain formulas and an expression of the new formula are provided in “Formulas characterizing stress strain curves” section. Test data of various types of steels were referred to in “Test data” section. “Validation and comparison” section validated the proposed formula with test data and comparisons with other formulas were also listed. Then, a limitation of the proposed formula is discussed in “Discussion” section. Finally principal conclusions are drawn in “Conclusion” section.

## Formulas characterizing stress strain curves

### Overview of existing formulas

The description of the stress–strain curves of metals by mathematical expressions has been a topic of research since the origin of classical mechanics. Numerous formulas have been proposed to describe the stress–strain curves. Osgood (1946) summarized 17 formulas used in the early age of study. Kleemola and Nieminen (1974) discussed the computational method of parameters for some commonly used formulas. Recently, existing common formulas have been reviewed and discussed by Hertelé et al. (2011).

The most well-known formulas are a series of simple formulas with a power function (Ludwik 1909; Ramberg and Osgood 1943; Hollomon 1945; Swift 1952; Hoffelner 2013). Among them, the Ramberg–Osgood formula (1943) has been widely accepted in the engineering field:

1 $$ \varepsilon_{p}\,=\, \left( {\frac{\sigma }{K}} \right)^{{\frac{1}{m}}} $$

Thus, the true stress–strain relationship can be expressed explicitly:

2 $$ \varepsilon\,=\, \frac{\sigma }{E} + \left( {\frac{\sigma }{K}} \right)^{{\frac{1}{m}}} $$

where *σ* is the true stress, *ε* is the true strain, *ε*_*p*_ is the plastic strain, E is the elastic modulus, and *K, m* are material parameters.

The convenience of this formula is that it can be easily linearized by taking logarithms of the true stress–plastic strain coordinates. Thus, the parameters can be obtained through linear regression analysis.

3 $$ m \cdot \log \varepsilon_{p} + \log K = \log \sigma $$

The deficiency of this formula is that it cannot characterize many materials in the full range exhibiting various distinct strain hardening stages, which have been observed in various types of steels (Quach et al. 2008; Rasmussen 2003; Abdella 2006; Bowen and Partridge 2002; Gardner and Nethercot 2004a, b) and other metals (Monteiro and Reed-Hill 1973; Markandeya et al. 2006). Therefore, many other types of formulas have been proposed (Ludwigson 1971; Voce 1948; Chinh et al. 2004). Ludwigson (1971) proposed such a formula, which accounts for the deviations at low strains by adding a second term to the Ludwik power law formula (Ludwik 1909):

4 $$ \sigma\,=\, K_{1} \cdot \varepsilon_{p}^{{m_{1} }} + e^{{K_{2} }} \cdot e^{{m_{2} \varepsilon_{p} }} $$

where *K*_1_, *m*_1_, *K*_2_, *m*_2_ are material parameters.

Compared to the Ramberg–Osgood formula, there is no single direct expression that shows a straight line in logarithmic or non-logarithmic coordinates. The formula shows a tendency toward linear behavior for large strains in a double-logarithmic stress–strain diagram. Therefore *K*_1_ and *m*_1_ can be obtained through linear regression of large strains. Thus, ∆ is defined as:

5 $$ \Delta\,=\,\sigma - K_{1} \cdot \varepsilon_{p}^{{m_{1} }} $$

*K*_*2*_ *, m*_*2*_ can be obtained through linear regression analysis of ln ∆ − *ε*_*p*_:

6 $$ \ln \Delta\,=\,{\text{K}}_{ 2}\,+\,{\text{m}}_{ 2} \cdot \varepsilon_{p} $$

The deficiency of this formula is also very clear: it cannot provide an explicit expression of *σ* − *ε* and could have difficulties in describing the smooth, gradual onset of yielding observed in many metallic materials (Hertelé et al. 2011).

Therefore, other formulas were proposed to characterize the full-range strain hardening behavior more accurately with segmented functions (Abdella 2006; Rasmussen 2003; Saab and Nethercot 1991; Hertelé et al. 2011; Real et al. 2014). Most of these formulas are material specific. Recently, Hertelé (2012a, b) proposed such an UGent formula to characterize the plastic behavior of pipeline steels.

7 $$ \varepsilon = \left\{ {\begin{array}{*{20}l} {\frac{\sigma }{E} + 0.002\left( {\frac{\sigma }{{\sigma_{0.2} }}} \right)^{{n_{1} }} } \hfill & {\sigma \le \sigma_{1} } \hfill \\ {\frac{\sigma }{E} + 0.002(\frac{\sigma }{{\sigma_{0.2} }})^{{n_{1} }} + 0.002\frac{{\sigma - \sigma_{1} }}{{\sigma_{2} - \sigma_{1} }}\left[ {\left( {\frac{\sigma }{{\sigma_{0.2} }}} \right)^{{n_{2} }} - \left( {\frac{\sigma }{{\sigma_{0.2} }}} \right)^{{n_{1} }} } \right] - \frac{0.002}{{\sigma_{2} - \sigma_{1} }}\left[ {\frac{{\sigma^{{n_{2} + 1}} { - }\sigma_{1}^{{n_{2} + 1}} }}{{(n_{2} + 1)\sigma_{0.2}^{{n_{2} }} }}{ - }\frac{{\sigma^{{n_{1} + 1}} { - }\sigma_{1}^{{n_{1} + 1}} }}{{(n_{1} + 1)\sigma_{0.2}^{{n_{1} }} }}} \right]} \hfill & {\sigma_{1} < \sigma < \sigma_{2} } \hfill \\ {\frac{\sigma }{E} + 0.002\left( {\frac{\sigma }{{\sigma_{0.2} }}} \right)^{{n_{2} }} - \frac{0.002}{{\sigma_{2} - \sigma_{1} }}\left[ {\frac{{\sigma_{2}^{{n_{2} + 1}} { - }\sigma_{1}^{{n_{2} + 1}} }}{{(n_{2} + 1)\sigma_{0.2}^{{n_{2} }} }}{ - }\frac{{\sigma_{2}^{{n_{1} + 1}} { - }\sigma_{1}^{{n_{1} + 1}} }}{{(n_{1} + 1)\sigma_{0.2}^{{n_{1} }} }}} \right]} \hfill & {\sigma \ge \sigma_{2} } \hfill \\ \end{array} } \right. $$

where *σ*_0.2_, *σ*_1_, *σ*_2_, *n*_1_, *n*_2_ are fitting parameters.

The UGent stress–strain model was developed to describe the strain hardening behavior of pipeline steels with two distinct stages. As listed in Eq. (7), for small plastic regions *σ* ≤ *σ*_1_, the UGent model respects a Ramberg–Osgood equation with a true 0.2 % proof stress *σ*_0.2_ and a first strain-hardening exponent *n*_1_; for large plastic region *σ* ≥ *σ*_*2*_, the UGent model respects a Ramberg–Osgood equation with the same 0.2 % proof stress *σ*_0.2_, but a possibly different strain-hardeing exponent *n*_2_; Between these two regions, there is a smooth transition where the curve shape gradually changes.

The deficiency of the UGent formula is that it is too complicated to apply in practice and the parameters are difficult to obtain.

### Proposed stress–strain formula

In order to deal with the deficiencies mentioned above, a new empirical formula is developed to describe the full-range strain hardening behavior of steels. The formula is based on the assumption that the real stress–strain curve tends to two different Ramberg–Osgood curves following the relationship of Eq. (8). It tends to the Ramberg–Osgood ε_p1_ − σ curve 1 by Eq. (9) in the small plastic strain region and Ramberg–Osgood ε_p2_–σ curve 2 by Eq. (10) in the large plastic strain region, respectively.

8 $$ \varepsilon_{p}\,=\,\frac{{\varepsilon_{p2} \cdot \exp (A\sigma + B) - \varepsilon_{p1} }}{1 + \exp (A\sigma + B)} $$

9 $$ \varepsilon_{p1}\,=\,\left( {\frac{\sigma }{{K_{1} }}} \right)^{{\frac{1}{{m_{1} }}}} $$

10 $$ \varepsilon_{p2}\,=\,\left( {\frac{\sigma }{{K_{2} }}} \right)^{{\frac{1}{{m_{2} }}}} $$

K_1_, K_2_, m_1_, m_2_, A, B are material fitting parameters.

**Fig. 1:**
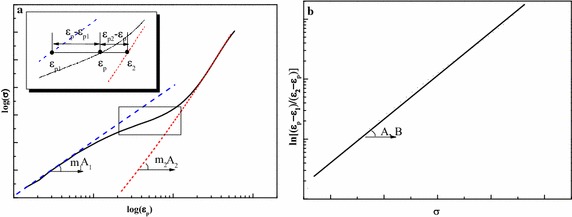
Graphical fitting procedure of the proposed formula. **a** Fitting procedure of m​_1_, A_1_ and m_2_, A_​2_. **b** Fitting procedure of *A*, *B*

The optimal parameter values of the proposed formula can be obtained through least-squares fitting method as depicted in Fig. 1 in following procedure:

- In the small scale yielding plastic area, a Ramberg–Osgood formula with m_1_, k_1_ is assumed to be followed, defined as ε_p1_-σ line in Fig. 1a. The parameters can be easily obtained through a linear regression analysis as Eq. (9) in the log(ε_p_) − log(σ) coordinate.
  11 $$ m_{1} \cdot \log \varepsilon_{p} + \log K_{1} = \log \sigma $$
- In the large scale yielding plastic area, a Ramberg–Osgood formula with m_2_, k_2_ should be followed, defined as ε_p2_ − σ line in Fig. 1a. The parameters can also be easily obtained through a linear regression analysis in the same way through Eq. (10):
  12 $$ m_{2} \cdot \log \varepsilon_{p} + \log K_{2} = \log \sigma $$
- In the transition between these two curves mentioned above, the ratio value of ε_p_ − ε_p1_ to ε_p2_ − ε against stress shows a linear relation of Eq. (13), in the coordinate depicted in Fig. 1b. The parameters A, B can be obtained through a linear regression analysis directly.
  13 $$ \ln \frac{{\varepsilon_{p} - \varepsilon_{p1} }}{{\varepsilon_{p2} - \varepsilon_{p} }} = A\sigma + B $$

## Test data

To validate the proposed formula, tensile tests at ambient temperature have been performed on three high strength steels. A strain rate of 5 × 10^−4^ s^−1^ was kept in loading to avoid any stress wave effect and to keep in a quasi-static mode. Test data of other steels done by Hertelé et al. (2011) were also selected. The basic tensile characteristics of the steels are summarized in Table 1. PCrNi3MoVA, G4335V, 32CrNi3MoVA are three high strength steels in China used for gun barrels, known as gun steels; API X70 is used for pipeline; TRIP 690 is a high strength Transformation Induced Plasticity steel; DIN 1.4462 is a stainless steel alloy.

**Table 1:** Tensile characteristics of the steels

Materials	Brand	Elastic modulus (MPa)	Yielding strength R_p0.2_ (MPa)	Tensile strength R_m_ (MPa)	R_p0.2_/R_m_	Uniform elongation
Gun steels	PCrNi3MoVA	215,000	962	1081	0.890	0.066
	G4335V	212,000	972	1160	0.838	0.068
	32CrNi3MoVA	201,000	985	1115	0.883	0.069
Pipeline steel	API X70	203,700	521	606	0.860	0.085
TRIP steel	TRIP 690	204,900	493	719	0.686	0.196
Stainless steel	DIN 1.4662	208,100	490	728	0.673	0.181

**Table 2:** Fitting parameters of other formulas

Formula	Parameters/dimension	G4335V	PCrNi3MoVA	32CrNi3MoVA	Pipeline steel	TRIP steel	Stainless steel
Ramberg–Osgood	K/MPa	1375	1293	1361	763	1015	1011
	m	0.0433	0.0482	0.0545	0.0642	0.1223	0.1234
Ludwigson	K_1_/MPa	1550	1467	1534	832	1180	1260
	m_1_	0.079	0.085	0.090	0.0915	0.181	0.212
	K_2_	5.010	5.39	4.540	4.42	5.12	4.80
	m_2_	−202.4	−296.3	−584.7	−230	−155	−38.1
Ugent	σ_0.2_/MPa	–	–	–	521	493	490
	n_1_	–	–	–	26.5	12.4	5.11
	n_2_	–	–	–	15.5	8.0	10.7
	σ_1_/MPa	–	–	–	536	535	490
	σ_2_/MPa	–	–	–	579	670	460
Gardner	n	–	–	–	15.1	16.5	4.43
	E_0.2_/10^3^ MPa	–	–	–	15.9	13.9	44.2
	n_0.2,1.0_^′^	–	–	–	1.55	2.20	3.05

**Fig. 2:**
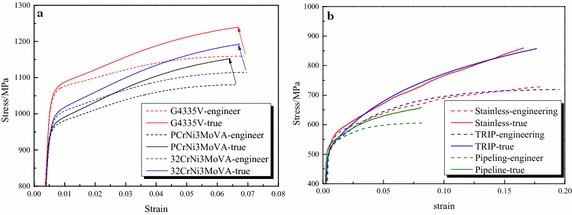
Measured stress–strain curves of the steels. **a** Gun steels. **b** Other steels

Figure 2 depicts the engineering and true stress–strain curves. The parts of the engineering stress–strain curves after necking were ignored and the true stress–strain curves were obtained through the well-known converting formulas *ɛ* = ln (1 + *ɛ*_*e*_) and *σ* = *σ*_*e*_(1 + *ɛ*_*e*_).

## Validation and comparison

The proposed formula has been applied to the test data of all the six steels. The optimal parameter values for each steel were obtained through the fitting procedure mentioned above (“Proposed stress–strain formula” section). The general Ramberg–Osgood formula (Ramberg and Osgood 1943), Ludwigson formula (Ludwigson 1971), UGent formula (Hertelé et al. 2011) and a material-specific Gardner formula (Gardner and Nethercot 2004a, b) have also been applied to the data for comparison.

Additionally, a difference approximation was conducted on the test data to obtain the strain hardening rate:

14 $$ \left( {\frac{d\sigma }{d\varepsilon }} \right)_{i} = \frac{{\sigma_{i + 1} - \sigma_{i} }}{{\varepsilon_{i + 1} - \varepsilon_{i} }} $$

**Fig. 3:**
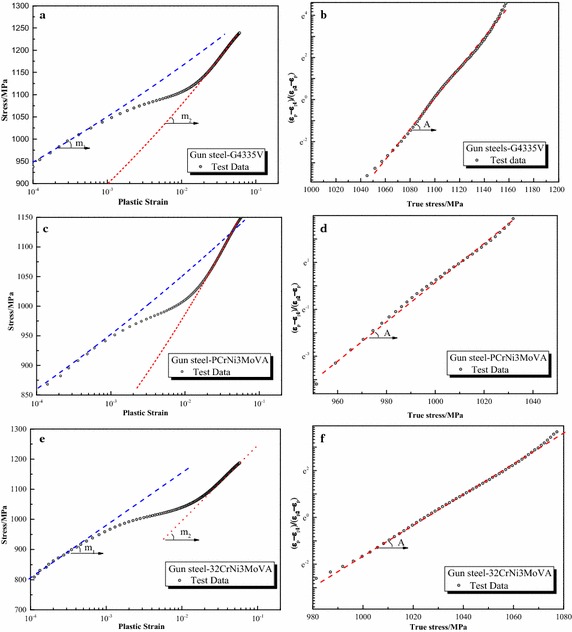
Graphical fitting procedure of the proposed formula for gun steels. **a** Fitting procedure of m_​1_, A_1_ and m_2_, A_​2_ for G4335V steel. **b** Fitting procedure of *A*, *B* for G4335V steel. **c** Fitting procedure of m_​1_, A_1_ and m_2_, A​_2_ for PCrNi3MoVa steel. **d** Fitting procedure of *A*, *B* for PCrNi3MoVa steel. **e** Fitting procedure of m_​1_, A_1_ and m_2_, A​_2_ for 32CrNi3MoVA steel. **f** Fitting procedure of *A*, *B* for 32CrNi3MoVA steel

**Fig. 4:**
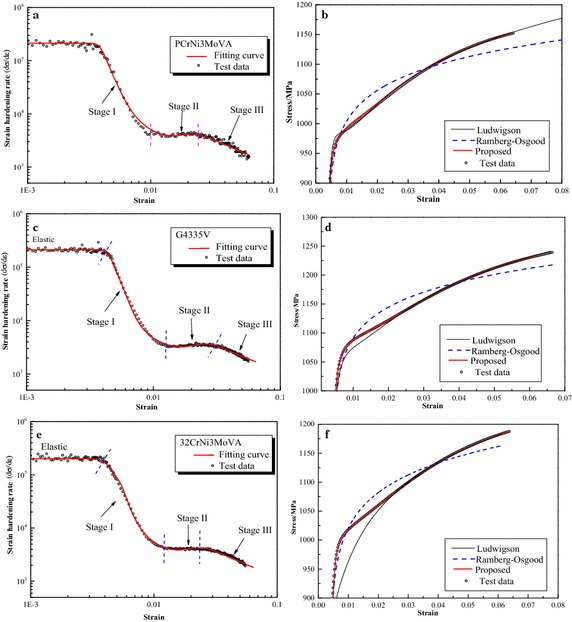
Fitting curves and test results of gun steels. **a** Strian hardening rate of PCrNi3MoVA. **b** Comparison of the fitting curves of PCrNi3MoVA. **c** Strain hardening rate of G4335V. **d** Comparison of the fitting curves of G4335V. **e** Strain hardening rate of 32CrNi3MoVA. **f** Strain hardening rate of 32CrNi3MoVA

**Fig. 5:**
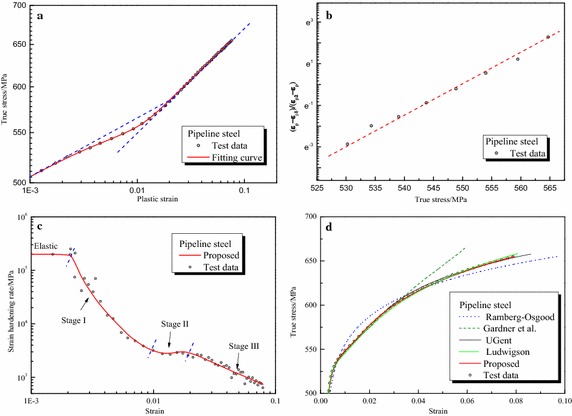
Fitting curves of proposed formula with the test results of pipeline steel. **a** Fitting procedure of m_1_, A_1_ and m_2_, A_2_. ​**b** Fitting procedure of *A*, *B*. **c** Strain hardening rate. **d** Comparison of the fitting curves

**Fig. 6:**
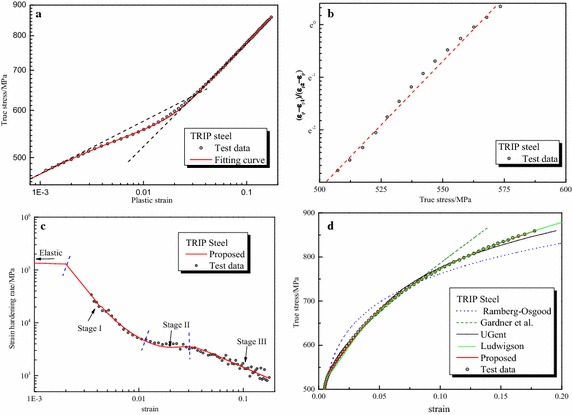
Fitting curves of proposed formula with the test results of TRIP steel. **a** Fitting procedure of m_1_, A_1_ and m_2_, A_2_. ​**b** Fitting procedure of *A*, *B*. **c** Strain hardening rate. **d** Comparison of the fitting curves

**Fig. 7:**
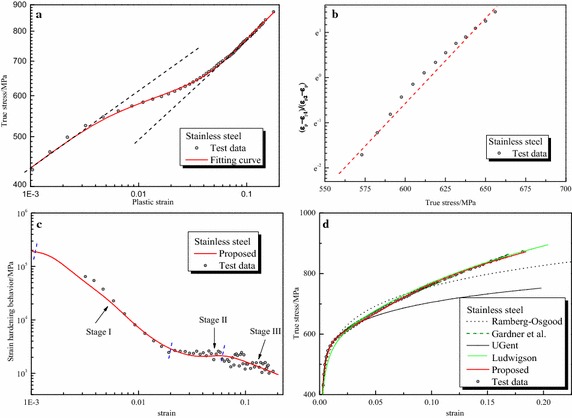
Fitting curves of proposed formula with the test results of stainless steel. **a** Fitting procedure of m_1_, A_1_ and m_2_, A_2_. ​**b** Fitting procedure of *A*, *B*. **c** Strain hardening rate. **d** Comparison of the fitting curves

Parameters of the proposed formula for all steels are summarized in Table 3 and other formulas in Table 2. Furthermore, Fig. 3 depicts the graphical fitting procedures for three gun steels. The strain hardening rate-strain curves and stress–strain curves for three gun steels are shown in Fig. 4. Figures 5, 6 and 7 depict the graphical fitting procedures (a, b), strain hardening rate-stress curves (c) and stress–strain curves (d) for pipeline steel, TRIP steel and stainless steel, respectively.

**Table 3:** Parameters of the proposed formula

Materials	Parameters of the proposed formula					
	A	B	K_1_ (MPa)	m_1_	K_2_ (MPa)	m_2_
G4335V	0.0715	−78.544	1429.39	0.0446	1549.67	0.0789
PCrNi3MoVA	0.0801	−80.675	1301.3	0.0457	1466.5	0.0845
32CrNi3MoVA	0.0631	−64.759	1630.76	0.0749	1534.41	0.0899
Pipeline steel	0.1414	−77.722	700.49	0.0471	815.27	0.0849
TRIP steel	0.0491	−27.643	897.02	0.0931	1173.82	0.1785
Stainless steel	0.0391	−24.016	1274.68	0.1558	1247.96	0.2088

It can be observed from those figures that: First, test data of all the steels show a three-stage hardening behavior which can be seen clearly in the strain hardening rate-strain coordinate. Stage I ends at approximately ε = 0.02 for stainless steel and ε = 0.01 for others; Stage II ends at roughly at ε = 0.06 for stainless steel and ε = 0.02 for others.

The difference in the strain hardening rate can be attributed to the operation of different deformation mechanisms (Kocks and Mecking 2003; Montazeri-Pour and Parsa 2016): Stage I exhibits a distinct decline hardening rate. The sudden drop of hardening rate is associated with cross-slip of dislocations bypassing the heads of piled up dislocations (Hockauf and Meyer 2010). After passing the initial Stage I, hardening rate decreases to another region with a constant value defined as Stage II. Stage II exhibits an almost constant hardening rate behavior which is contributed to a steady state for storage and annihilation of dislocations (Zehetbauer and Seumer 1993). After Stage II, the hardening rate decreases continuously into a separate Stage III up to necking point (Kocks and Mecking 2003). Features of Stage III are analogous to Stage I and are considered to be connected with point defect generation and absorption (Zehetbauer and Seumer 1993).

Second, linear relationship assumed in the fitting procedure of the proposed formula is verified for all the test data. The proposed formula provides satisfactory representations of the test data for all the six steels in the full range. It can characterize excellently the three-stage strain hardening behavior of steels observed in the test. Six parameters of the formula, all of which are easy to understand and interpret in an intuitive way, can be obtained directly and easily through linear regression.

Third, for other formulas, it can be found that: The Ludwigson formula generally seems to provide accurate description of all curves for large plastic strain, e.g. Stage III, but lacks accuracy at a lower strain, below 0.02 for gun steels and stainless steel. This formula also cannot be utilized directly because there is no explicit expression of strain. The Gardner formula, on the other hand, seems to provide an accurate description of the full range curve for stainless steel and the lower strain parts for pipeline steel up to 0.035 and TRIP steel up to 0.07. The UGent formula provides an accurate description of pipeline steel and TRIP steel up to plastic regions near necking but lack accuracy for stainless steel. The fitting procedure of UGent formula is cumbersome and some parameters are arbitrary.

## Discussion

Limitations of the proposed formula are discussed in this section. First, obviously the proposed formulas cannot be utilized to describe the strain hardening behavior of steels with a sharp or specific yielding strength, which can be observed in some carbon steels.

Second, as mentioned in “Test data” section, in this paper the strain hardening response of materials is characterized by the stress–strain curves documented in tensile tests. The parts of the engineering stress–strain curves after necking were ignored due to the local necking effect. However, when extremely large deformation was mentioned, this procedure is not quite enough.

Third, to simplify the loading condition, quasi-static loading mode is considered in this paper. However, it is well known that temperature and strain rate have great effect on the plastic deformation behavior. More works are needed on these issues.

## Conclusion

In the present paper, a new formula has been proposed to describe the full range strain hardening behavior of steels. The test results demonstrate that the test data of all the six steels observed have a three-stage hardening behavior. The proposed formula, based on two different Ramberg–Osgood formulas, can characterize such behavior in the full range using a single expression. The parameters of the formula can be easily and directly obtained through linear regression analysis. The fitting curves and test results were identified to have excellent agreement for all the six steels.
