# Erratum to: Description of full-range strain hardening behavior of steels

**DOI:** 10.1186/s40064-016-3785-x

**Published:** 2016-12-22

**Authors:** Tao Li, Jinyang Zheng, Zhiwei Chen

**Affiliations:** 1 Institute of Process Equipment, Zhejiang University, Hangzhou, 310027 Zhejiang People’s Republic of China; 2 Chinese Standardization Committee on Boilers and Pressure Vessels, Beijing, 100029 People’s Republic of China; 3 High-Pressure Process Equipment and Safety Engineering Research Center of Ministry of Education, Zhejiang University, Hangzhou, 310027 Zhejiang People’s Republic of China; 4 The State Key Laboratory of Fluid Power Transmission and Control, Zhejiang University, Hangzhou, 310027 Zhejiang People’s Republic of China

## Erratum to: SpringerPlus (2016) 5:1316 DOI 10.1186/s40064-016-2998-3

Following the publication of our article (Li et al. 2016), we have become aware that the legends of Fig. 2 and Table 2 should read as follows:

Fig. 2 Measured stress–strain curves of the steels. **a** Gun steels. **b** Other steels. **b** is adapted from Figure 10 in *International Journal of Nonlinear Mechanics*, Vol 46(3), Hertelé S, De Waele W, Denys R, A generic stress–strain model for metallic materials with two-stage strain hardening behaviour, pp 519–531, Copyright (2010) with permission of Elsevier (Hertelé et al. 2011).

Table 2 Fitting parameters of other formulas. Data for pipeline steel, TRIP steel and stainless steel are from Table 3 in *International Journal of Nonlinear Mechanics*, Vol 46(3), Hertelé S, De Waele W, Denys R, A generic stress–strain model for metallic materials with two-stage strain hardening behaviour, pp 519–531, Copyright (2010) adapted with permission of Elsevier (Hertelé et al. 2011).
